# DECIMER: towards deep learning for chemical image recognition

**DOI:** 10.1186/s13321-020-00469-w

**Published:** 2020-10-27

**Authors:** Kohulan Rajan, Achim Zielesny, Christoph Steinbeck

**Affiliations:** 1 Institute for Inorganic and Analytical Chemistry, Friedrich-Schiller-University Jena, Lessingstr. 8, 07743 Jena, Germany; 2 Institute for Bioinformatics and Chemoinformatics, Westphalian University of Applied Sciences, August-Schmidt-Ring 10, 45665 Recklinghausen, Germany

**Keywords:** Optical chemical entity recognition, Chemical structure, Deep learning, Deep neural networks, Autoencoder/decoder

## Abstract

The automatic recognition of chemical structure diagrams from the literature is an indispensable component of workflows to re-discover information about chemicals and to make it available in open-access databases. Here we report preliminary findings in our development of Deep lEarning for Chemical ImagE Recognition (DECIMER), a deep learning method based on existing show-and-tell deep neural networks, which makes very few assumptions about the structure of the underlying problem. It translates a bitmap image of a molecule, as found in publications, into a SMILES. The training state reported here does not yet rival the performance of existing traditional approaches, but we present evidence that our method will reach a comparable detection power with sufficient training time. Training success of DECIMER depends on the input data representation: DeepSMILES are superior over SMILES and we have a preliminary indication that the recently reported SELFIES outperform DeepSMILES. An extrapolation of our results towards larger training data sizes suggests that we might be able to achieve near-accurate prediction with 50 to 100 million training structures. This work is entirely based on open-source software and open data and is available to the general public for any purpose.

## Main text

**Fig. 1:**
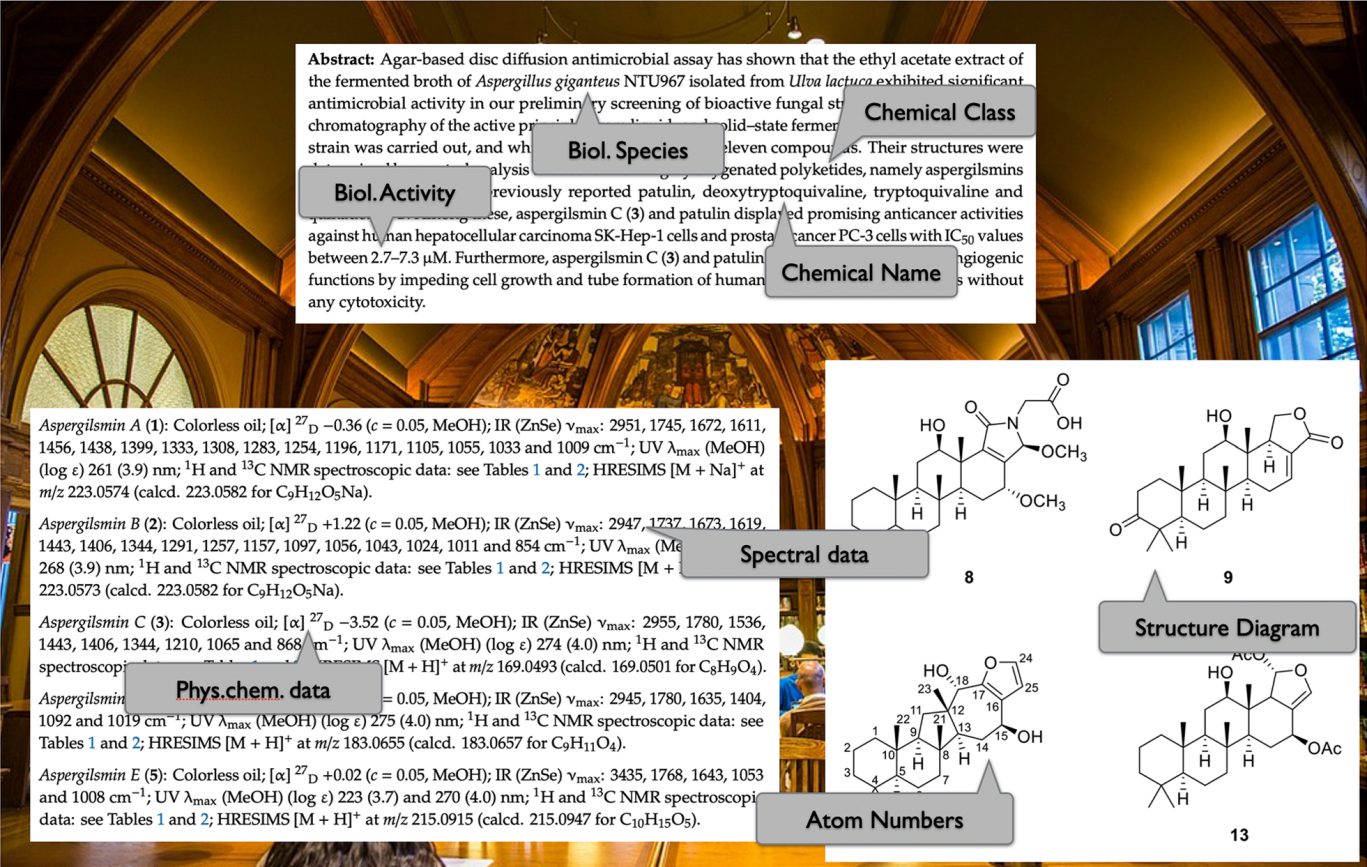
Information about a natural product is scattered across the various sections of an individual scientific article. Grouped around a structure and a chemical name, further information such as chemical classes, species, and organism parts from which the compound was isolated, spectral and other data are listed. Background image © Alina Chan, distributed under https://creativecommons.org/licenses/by-sa/4.0/deed.en, figures and text from Kwon et al. [12]

The automatic recognition of chemical structure diagrams from the chemical literature (herein termed Optical Chemical Entity Recognition, OCER) is an indispensable component of workflows to re-discover information about chemicals and to make it available in open-access databases. While the chemical structure is often at the heart of the findings reported in chemical articles, further information about the structure is present either in textual form or in other types of diagrams such as titration curves, spectra, etc. (Fig. 1).

Previous software systems for OCER have been described and were both incorporated into commercial and open-source systems. These software systems include Kekulé [1, 2], the Contreras system [3], the IBM system [4], CLIDE [5] as well as the open-source approaches chemOCR [6–8], ChemReader [9], OSRA [10] and ChemRobot described in a patent [11].

All of these software packages share a general approach to the problem, comprising the steps (a) scanning, (b) vectorization, (c) searching for dashed lines and dashed wedges, (d) character recognition, (e) graph compilation, (f) post-processing, (g) display and editing.

Each of the steps in such systems needs to be carefully hand-tuned both individually as well as for its interplay with the other steps. The incorporation of new image features to be detected is a laborious process.

We were recently inspired by the stunning success of AlphaGo Zero [13], a deep neural network (NN) based approach that enabled AlphaGo Zero to reach superhuman strength in the Game of Go by playing a potentially unlimited number of games against itself, starting with no more knowledge than the basic rules of the game. In this example, as well as in other prominent examples of successful deep learning, the key to success was the availability of a potentially unlimited or very large amount of training data.

The example of AlphaGo Zero made us realize that we are in a similar situation for the visual computing challenge described above. Instead of working with a necessarily small corpus of human-annotated examples from the printed literature, as has been common in the text mining and machine learning applications in chemistry in the past, we realised that we could generate training data from a practically unlimited source of structures generated by structure generators or by using the largest collections of open chemical data available to mankind.

After we started our work presented here, other attempts to use deep learning for OCER were reported. Work by the Schrödinger group [14] reports the successful extraction of machine-readable chemical structures from bitmaps but no software system available for the general public to replicate the reported results. A method called Chemgrapher [15] suggests to deal with the problem in a modular fashion by using a segmentation algorithm to segment the images containing chemical graphs to detect atoms locations, bonds and charges separately, and employ a graph building algorithm to re-generate the chemical graph.

**Fig. 2:**
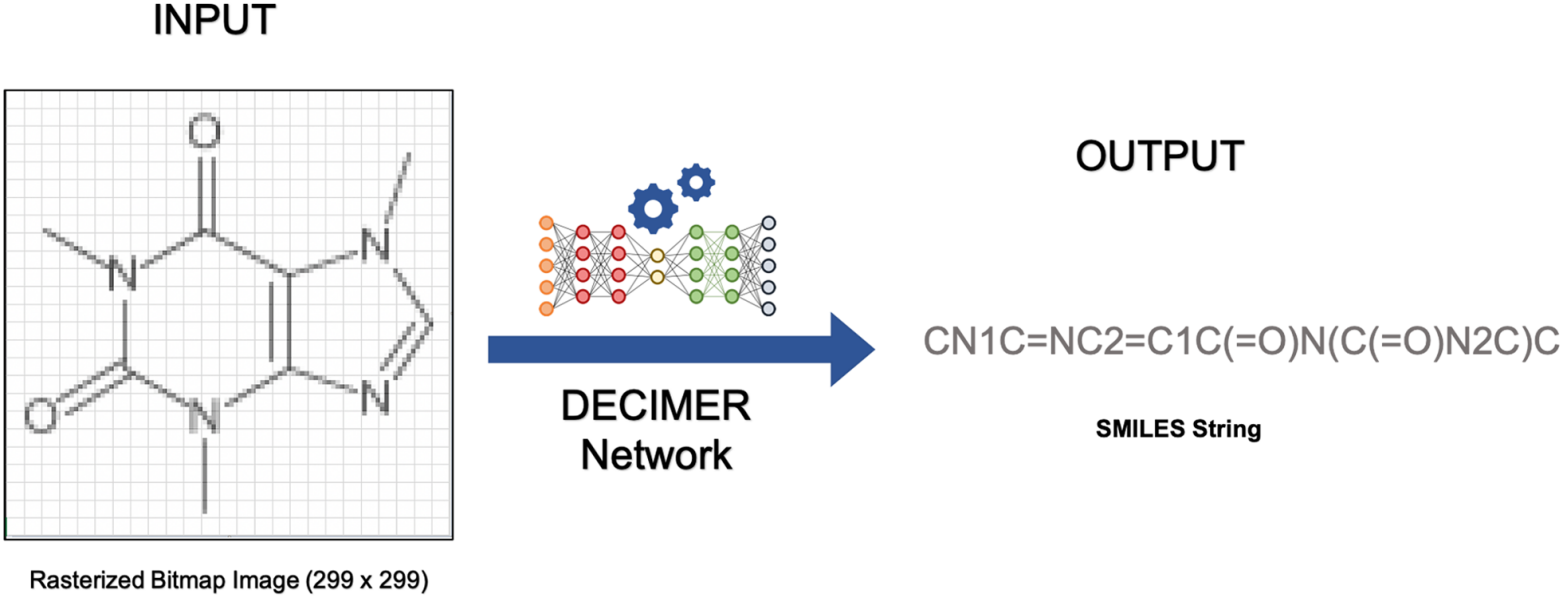
Chemical image to SMILES translation using DECIMER

Here we report preliminary findings of our development of Deep lEarning for Chemical ImagE Recognition (DECIMER), a deep learning method based on existing show-and-tell deep neural networks, which translates a pure bitmap image of a molecule, as found in publications, into a SMILES (Fig. 2). Unlike for example Chemgrapher, it makes no prior assumptions, such as the existence of bonds or element symbols in the graphic, about the structure of the underlying problem.

The training state reported here does not yet rival the performance of existing traditional approaches, but we present evidence that, given sufficient training data, our method will reach a comparable detection power without the need of the sophisticated engineering steps of an OCER workflow.

The principal idea reported here is to repurpose a show-and-tell deep NN designed for general photo annotation earlier and train it to report a series of SMILES tokens when presented with the bitmap of a chemical structure image. The original NN reported sentences like “A giraffe standing in a forest with trees in the background” when presented with a corresponding photo.

Instead of abstracting chemical diagrams from the chemical literature to generate training data, we decided to use structure diagram generators (SDG) like the one found in the Chemistry Development Kit (CDK) [16] to generate a potentially unlimited amount of training data. This type of training data can be accommodated to become more realistic and comparable to the varying picture quality in the chemical literature by using image manipulation such as blurring, adding noise, etc. As a source of input structures for the CDK SDG, we turned to PubChem [17], one of the largest databases of organic molecules. The following rules were used to curate the Pubchem data for our work presented here (in future versions of this deep NN, these rules might be relaxed):

- Molecules must,
- Have a molecular weight of fewer than 1500 Daltons,
- Not possess counter ions,
- Only contain the elements C, H, O, N, P, S, F, Cl, Br, I, Se and B,
- Not contain isotopes of Hydrogens (D, T),
- Have 5–40 bonds,
- Not contain any charged groups,
- Only contain implicit hydrogens, except in functional groups,
- Have less than 40 SMILES characters.

The generation of molecular bitmap images from chemical graphs was performed using the CDK SDG, which generates production quality 2D depictions to feed the deep learning algorithm. One random rotation for each molecule was used. No further modifications, such as the addition of noise, were applied. These types of modifications will be explored once a mature model has been reached.

The text data used here were SMILES [18] strings, which were encoded into different formats, regular SMILES, DeepSMILES [19] and SELFIES [20], to test the dependency of the learning success on the data representation. These datasets were used in different training models in order to evaluate their performance for our use case. With two dataset sizes, we confirmed the superiority of the DeepSMILES over the SMILES representation and continued to use DeepSMILES exclusively.

**Fig. 3:**
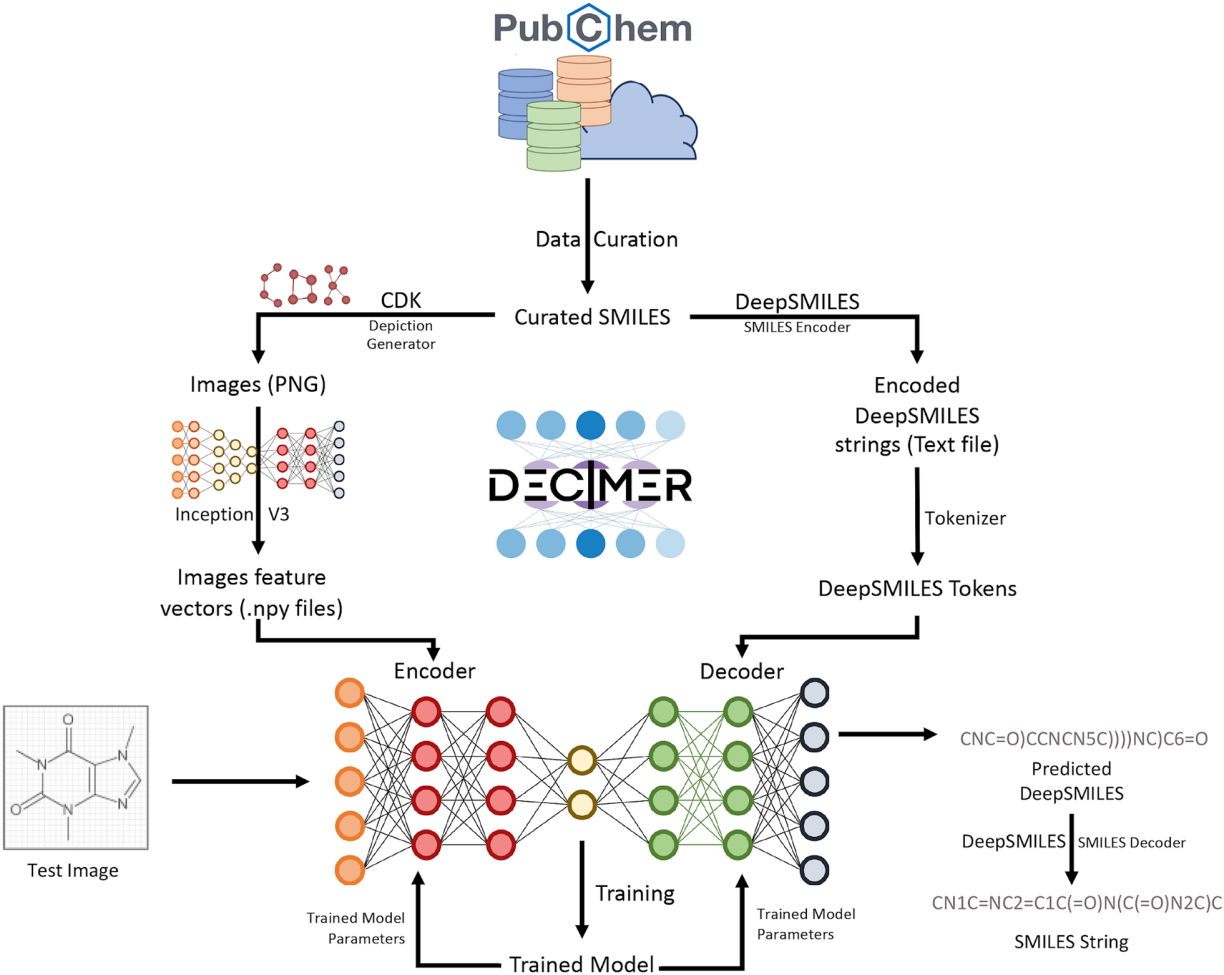
Schema of the DECIMER workflow

For our model (Fig. 3), we employed an autoencoder-based network with TensorFlow 2.0 [21] at the backend. This kind of network refers to the model designed by Xu et al. [22], in their work on Show, Attend and Tell, where they demonstrate a higher accuracy for an Image caption generation system with the attention mechanism. The TensorFlow team used these results for their implementation of Show, Attend and Tell, published at [23], which is used unaltered by us. Their encoder network is a convolutional NN (CNN), which consists of a single fully connected layer and a RELU activation function. Their decoder network is a recurrent NN (RNN), consisting of a gated recurrent unit (GRU) and two fully connected layers. The soft attention mechanism used in [23] was introduced by Bahdanau et al. [24].

We trained the model with DeepSMILES textual data and the corresponding bitmap of the chemical diagram. The text file is read by the model, the DeepSMILES is tokenized by the tokenizer, and the unique tokens are stored. The images are converted into feature vectors by using the unaltered Inception V3 [25] model and saved as NumPy arrays.

The model accuracy is determined by the average of all the calculated Tanimoto similarity scores as well as the number of Tanimoto 1.0 hits. The Tanimoto coefficient is superior to simple structure isomorphism because it yields the improvement of the recognition even when identity is not (yet) reached, and with a low degeneracy fingerprint such as the Pubchem fingerprint used here, the Tanimoto 1.0 is almost identical to the more rigorous structure isomorphism.

Initially, we trained multiple models with small training datasets to obtain the best hyper-parameters for our network. Exploration of the hyperparameter space led to 640 images per batch size, with embedding dimension size of 600 for the images, which we depicted on a 299 × 299 canvas size to match the Inception V3 model. We used an Adam optimizer with a learning rate 0.0005 and Sparse Categorical Cross entropy to calculate the loss. We trained all the models for 25 epochs, which typically led to convergence. Once the models converged, we started the evaluation of the test set.

The models were trained on an inhouse server equipped with an nVidia Tesla V100 Graphics Card, 384 GB of RAM and two Intel(R) Xeon(R) Gold 6230 CPUs. Even though the training entirely happens on the GPU, the initial dataset preparation was CPU-based.

**Fig. 4:**
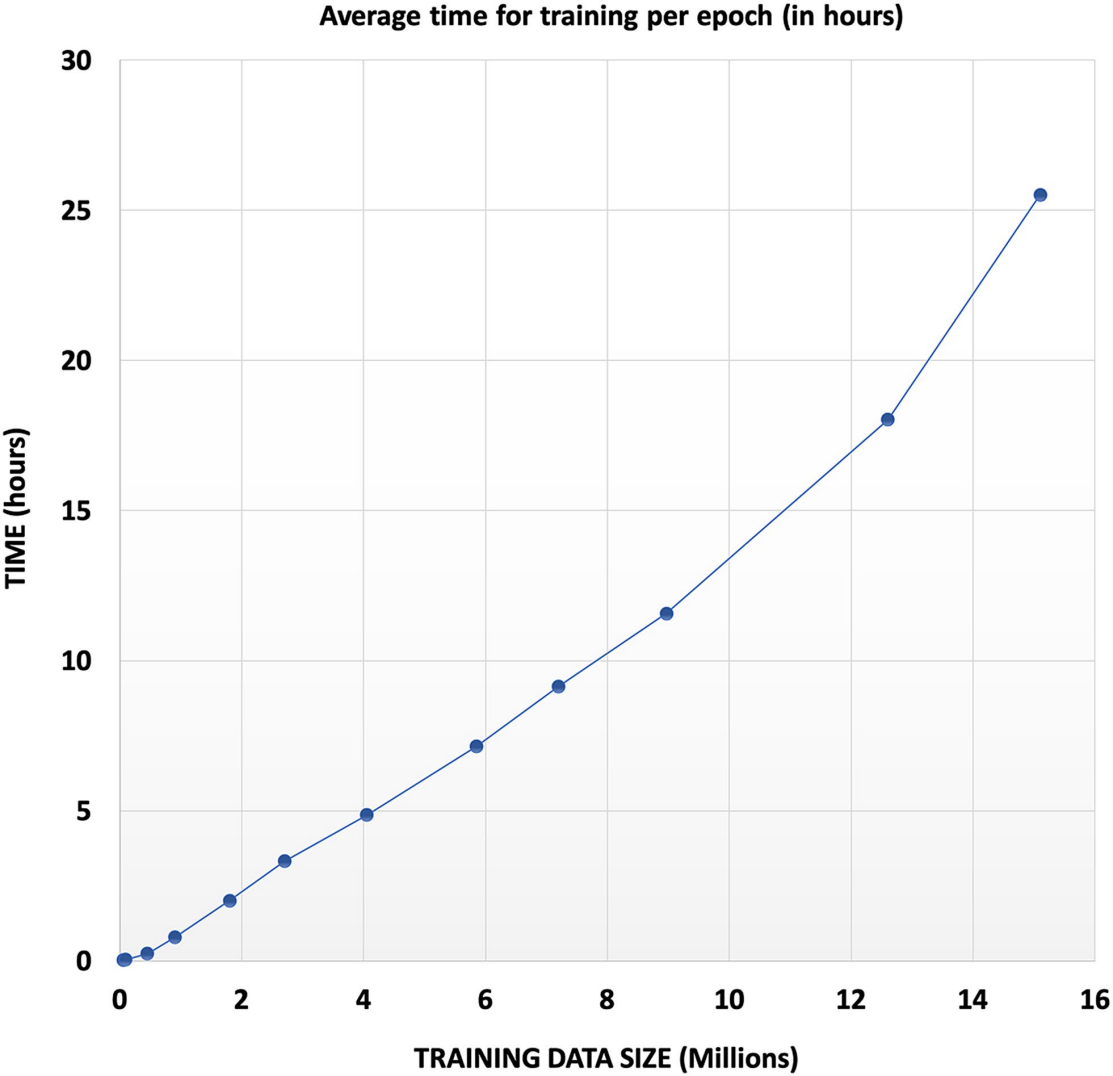
Average time spent on training each epoch with increasing dataset size

Training time obviously scales with data size (Table 1, Fig. 4). Model success was evaluated with an independent test data set. During the preparation of this manuscript, initial experiments with parallel training indicated that scaling was not satisfactory beyond 2 or 3 GPUs.

**Table 1:** Dataset sizes used in this work with corresponding computing times

Dataset index	Train data size	Test data size	Avrg. time/epoch (s)	Time for 25 epochs (s)
1	54,000	6000	94.32	2358
2	90,000	10,000	159.88	3997
3	450,000	50,000	880.6	22,015
4	900,000	100,000	2831.8	70,795
5	1,800,000	200,000	7239.28	180,982
6	2,700,000	300,000	11,964.72	299,118
7	4,050,000	450,000	17,495.12	437,378
8	5,850,000	650,000	25,702	642,550
9	7,200,000	800,000	32,926.8	823,170
10	8,969,751	996,639	41,652.24	1,041,306
11	12,600,000	1,400,000	64,909.28	1,622,732
12	15,102,000	1,678,000	91,880.84	2,297,021

The time for training the model with 15 million structures corresponds to approximately a month on a single Tesla V100 GPU

**Fig. 5:**
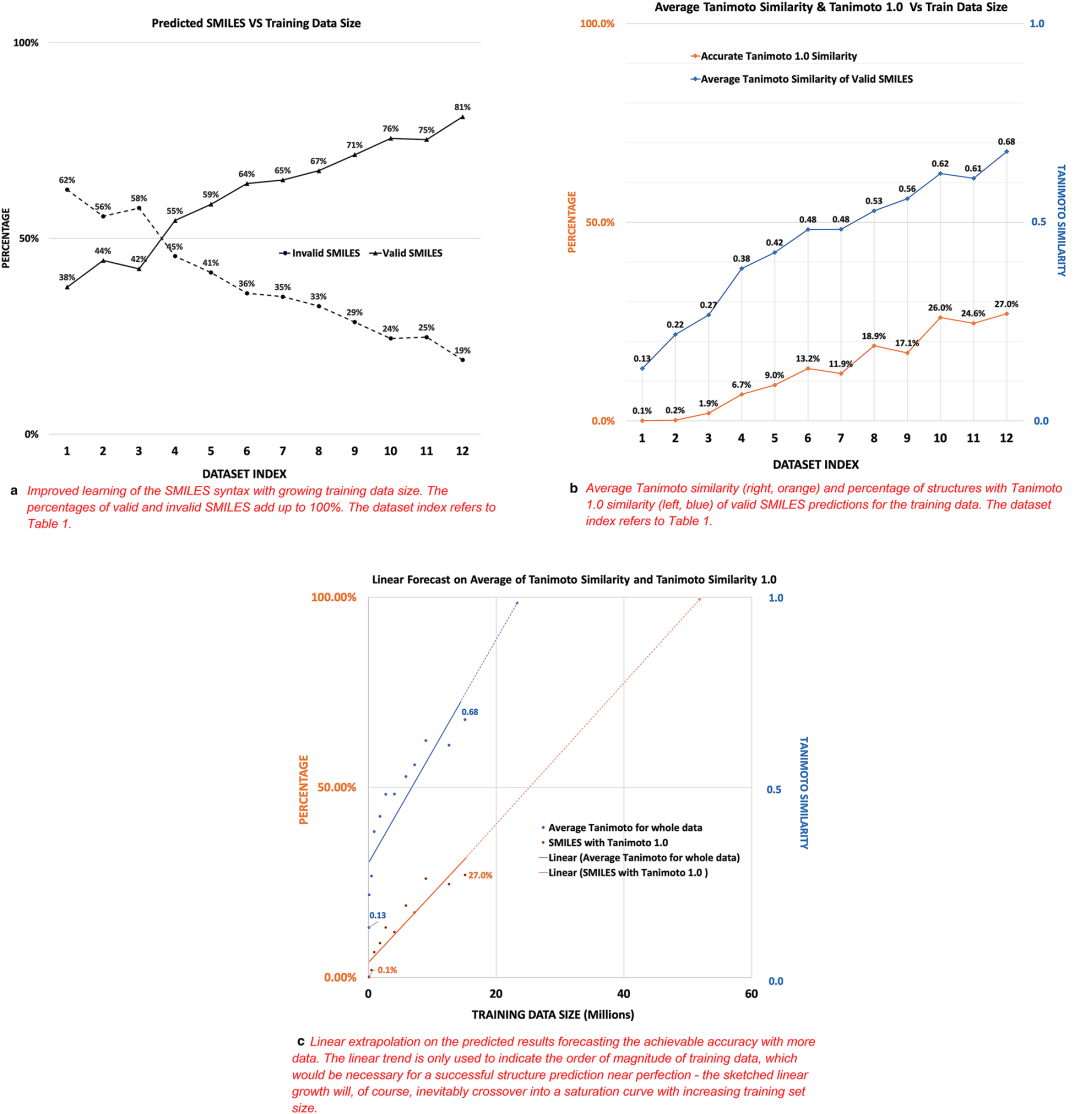
Development of training success indicators with increasing train data size. **a** Improved learning of the SMILES syntax with growing training data size. The percentages of valid and invalid SMILES add up to 100%. The dataset index refers to Table 1. **b** Average Tanimoto similarity (right, orange) and percentage of structures with Tanimoto 1.0 similarity (left, blue) of valid SMILES predictions for the training data. The dataset index refers to Table 1. **c** Linear extrapolation on the predicted results forecasting the achievable accuracy with more data. The linear trend is only used to indicate the order of magnitude of training data, which would be necessary for a successful structure prediction near perfection—the sketched linear growth will, of course, inevitably crossover into a saturation curve with increasing training set size

**Fig. 6:**
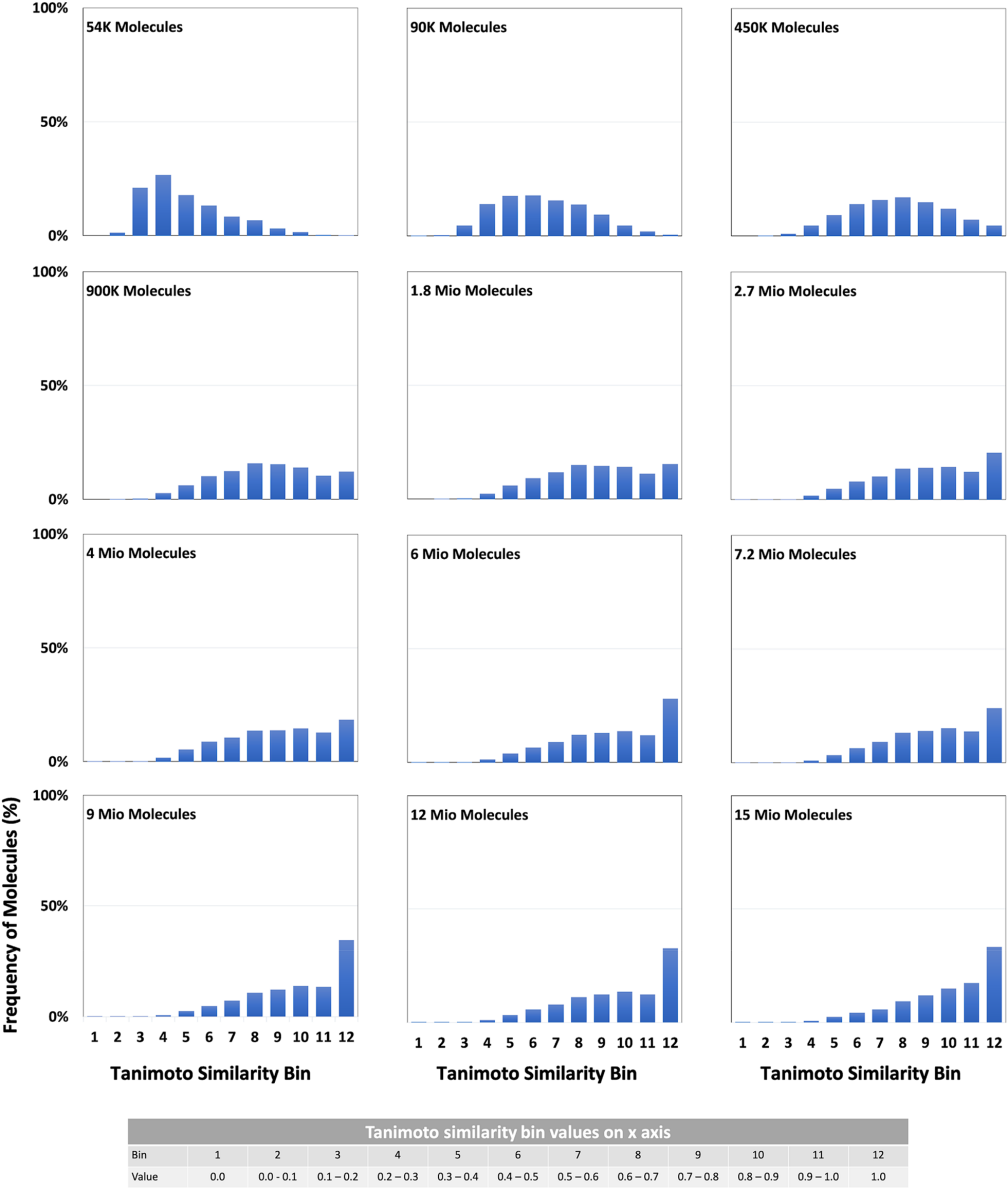
Distribution of Tanimoto-Similarity between the training structures and the structure recognised by DECIMER. Y-axis: frequency of molecules in percentage, x-axis: Tanimoto similarity range in bins

Here we report our results for training data sizes between 54,000 and 15,000,000 structures, with the largest training data set taking 27 days to converge on the hardware reported above (Table 1, Fig. 4). Figures 5 and 6 show the growth of the accuracy of predictions with increasing train data size.

Training success was determined with a number of indicators (Figs. 5 and 6), such as the percentage of Tanimoto 1.0 predictions, the average Tanimoto similarity of all predictions, and the percentage of invalid SMILES produced by the model. Figure 5a demonstrates that the model’s ability to produce valid SMILES and avoid invalid ones steeply increases with larger training datasets. The same can be observed for the two key parameters of this application, the average Tanimoto similarity and the Tanimoto 1.0 percentage, which indicate the fitness of the model to accurately generate a machine-readable structure from a bitmap of a chemical diagram. We show here that the similarity of predicted chemical graphs to the correct chemical graph becomes constantly better with more training data. While we regularly operate with the chemical paradigm that similar structures have similar properties and therefore increasing structure similarity would convey increasing similarity of inherent properties, we chose to confirm that this would hold in our application case. We therefore further evaluated the models’ success with additional descriptors such as LogP or ring count between original and the predicted SMILES, which indicates that the model consistently produces better and better machine representations with growing training data size. The improvements do not seem to converge prematurely.

In order to assess the promise of these preliminary results, we performed an idealistic linear extrapolation of our data toward larger training data sizes, which indicate that close-to-perfect detection of chemical structures would require training data sizes with 50 to 100 million structures. Such a training data volume will likely require a training time of 4 months with our setup with a single GPU. We are currently experimenting with the distributed learning solution currently available in the Tensorflow 2.0 API to reduce this training time significantly, also evaluating Google’s Tensor Processing Units (TPU).

## Conclusions

Here we have presented preliminary results indicating that a show-and-tell deep neural network setup has the potential to successfully extract a machine-readable structure representation when trained with tens of millions of examples. The training setup makes minimal assumptions about the problem. Training success depended on the input data representation. DeepSMILES were superior over SMILES and we have the preliminary indication that the recently reported SELFIES outperform DeepSMILES. For example, for a training data size of 6 Mio images, we obtained an average Tanimoto similarity of 0.53 with DeepSMILES and 0.78 with SELFIES. An extrapolation of our results towards larger training data sizes suggests that we might be able to achieve near-accurate prediction with 50 to 100 million training structures. Such training can be completed in uncomfortable but feasible time spans of several months on a single GPU.

Our work is entirely based on open-source software and open data and is available to the general public for any purpose.

We are currently moving towards larger training sets with the use of parallelization and more powerful hardware and hope to report the results in a full paper on this work in due time.

## Data Availability

The code for DECIMER is available at https://github.com/Kohulan/DECIMER, https://github.com/Kohulan/DECIMER-Image-to-SMILES.

## References

[1] McDaniel JR, Balmuth JR (1992). Kekule: OCR-optical chemical (structure) recognition. J Chem Inf Model.

[2] Borman S (1992). New computer program reads, interprets chemical structures. Chem Eng News.

[3] Contreras ML, Allendes C, Alvarez LT, Rozas R (1990). Computational perception and recognition of digitized molecular structures. J Chem Inf Model.

[4] Casey R, Boyer S, Healey P, Miller A, Oudot B, Zilles K (1993) Optical recognition of chemical graphics. In: Proceedings of 2nd international conference on document analysis and recognition (ICDAR ’93). IEEE Computer Society Press, Washington, DC, pp 627–631. https://ieeexplore.ieee.org/document/395658/

[5] Ibison P, Jacquot M, Kam F, Neville AG, Simpson RW, Tonnelier C (1993). Chemical literature data extraction: the CLiDE Project. J Chem Inf Model.

[6] Zimmermann M, Bui Thi LT, Hofmann M (2005) Combating illiteracy in chemistry: towards computer-based chemical structure reconstruction. ERCIM News 60(60):40–41. https://www.ercim.eu/publication/Ercim_News/enw60/zimmermann.html, https://www.researchgate.net/publication/228766116_Combating_illiteracy_in_chemistry_towards_computer-based_chemical_structure_reconstruction

[7] Algorri M-E, Zimmermann M, Friedrich CM, Akle S, Hofmann-Apitius M (2007) Reconstruction of chemical molecules from images. In: 2007 29th annual international conference of the IEEE engineering in medicine and biology society. IEEE, New York, pp 4609–4612. https://ieeexplore.ieee.org/document/4353366/10.1109/IEMBS.2007.4353366 18003032

[8] Algorri M-E, Zimmermann M, Hofmann-Apitius M (2007) Automatic recognition of chemical images. In: Eighth Mexican international conference on current trends in computer science (ENC 2007). IEEE, New York, pp 41–46. https://ieeexplore.ieee.org/document/4351423/

[9] Park J, Rosania GR, Shedden KA, Nguyen M, Lyu N, Saitou K (2009). Automated extraction of chemical structure information from digital raster images. Chem Cent J.

[10] Filippov IV, Nicklaus MC (2009). Optical structure recognition software to recover chemical information: OSRA, an open source solution. J Chem Inf Model.

[11] Karthikeyan M (2017) Chemical structure recognition tool. US Patent 9,558,403 B2

[12] Kwon O-S, Kim D, Kim C-K, Sun J, Sim CJ, Oh D-C (2020). Cytotoxic scalarane sesterterpenes from the sponge *Hyrtios erectus*. Mar Drugs.

[13] Silver D, Schrittwieser J, Simonyan K, Antonoglou I, Huang A, Guez A (2017). Mastering the game of go without human knowledge. Nature.

[14] Staker J, Marshall K, Abel R, McQuaw CM (2019). Molecular structure extraction from documents using deep learning. J Chem Inf Model.

[15] Oldenhof M, Arany A, Moreau Y, Simm J (2020) ChemGrapher: optical graph recognition of chemical compounds by deep learning. https://arxiv.org/abs/2002.09914 10.1021/acs.jcim.0c00459 32924466

[16] Willighagen EL, Mayfield JW, Alvarsson J, Berg A, Carlsson L, Jeliazkova N (2017). The Chemistry Development Kit (CDK) v2.0: atom typing, depiction, molecular formulas, and substructure searching. J Cheminform.

[17] Kim S, Chen J, Cheng T, Gindulyte A, He J, He S (2019). PubChem 2019 update: improved access to chemical data. Nucleic Acids Res.

[18] Weininger D (1988). SMILES, a chemical language and information system: 1: introduction to methodology and encoding rules. J Chem Inf Comput Sci.

[19] O’Boyle N, Dalke A (2018). chemRxiv.

[20] Krenn M, Häse F, Nigam A, Friederich P, Aspuru-Guzik A (2020) Self-referencing embedded strings (SELFIES): a 100% robust molecular string representation. https://github.com/aspuru-guzik-group/selfies. Accessed 2 June 2020

[21] Abadi M, Agarwal A, Barham P, Brevdo E, Chen Z, Citro C et al (2016) TensorFlow: large-scale machine learning on heterogeneous distributed systems. https://arxiv.org/abs/1603.04467

[22] Xu K, Ba JL, Kiros R, Cho K, Courville A, Salakhutdinov R et al (2015) Show, attend and tell: neural image caption generation with visual attention. In: 32nd International conference on machine learning, ICML 2015, vol 3, pp 2048–2057

[23] tensorflow. tensorflow/docs. https://github.com/tensorflow/docs/blob/master/site/en/tutorials/text/image_captioning.ipynb. Accessed 18 Aug 2020

[24] Bahdanau D, Cho KH, Bengio Y (2015) Neural machine translation by jointly learning to align and translate. In: 3rd international conference on learning representations, ICLR 2015—Conf Track Proc, pp 1–15

[25] Szegedy C, Vanhoucke V, Ioffe S, Shlens J, Wojna Z (2016) Rethinking the inception architecture for computer vision. In: Proceedings of the IEEE computer society conference on computer vision and pattern recognition, pp 2818–2826

